# Development and characterization of probiotic mucilage based edible films for the preservation of fruits and vegetables

**DOI:** 10.1038/s41598-021-95994-5

**Published:** 2021-08-16

**Authors:** Seyed Mohammad Davachi, Neethu Pottackal, Hooman Torabi, Alireza Abbaspourrad

**Affiliations:** 1 Department of Food Science, College of Agriculture and Life Sciences, Cornell University, Stocking Hall, Ithaca, NY 14853 USA; 2 Department of Materials Science and Engineering, College of Engineering, Cornell University, Bard Hall, Ithaca, NY 14853 USA

**Keywords:** Materials science, Engineering, Health care

## Abstract

There is growing interest among the public and scientific community toward the use of probiotics to potentially restore the composition of the gut microbiome. With the aim of preparing eco-friendly probiotic edible films, we explored the addition of probiotics to the seed mucilage films of quince, flax, and basil. These mucilages are natural and compatible blends of different polysaccharides that have demonstrated medical benefits. All three seed mucilage films exhibited high moisture retention regardless of the presence of probiotics, which is needed to help preserve the moisture/freshness of food. Films from flax and quince mucilage were found to be more thermally stable and mechanically robust with higher elastic moduli and elongation at break than basil mucilage films. These films effectively protected fruits against UV light, maintaining the probiotics viability and inactivation rate during storage. Coated fruits and vegetables retained their freshness longer than uncoated produce, while quince-based probiotic films showed the best mechanical, physical, morphological and bacterial viability. This is the first report of the development, characterization and production of 100% natural mucilage-based probiotic edible coatings with enhanced barrier properties for food preservation applications containing probiotics.

Throughout the past decade, the human microbiome has received increasing attention in both the medical and scientific community as well as the general public. The microbiome consists of the bionetwork of commensal, symbiotic, and pathogenic microorganisms residing in the body^1^. The number of bacteria inhabiting the human body is reported to be one order of magnitude more than human cells, the majority of which are primarily found in the gastrointestinal tract^2^. Several promising findings have underlined the importance of gut microbiota in protecting against invading pathogens and the regulation of various physiological functions such as metabolism, development, and stability of the immune and nervous system which, overall, results in prevention of inflammation and reduction in risks to conditions and diseases such as diarrhea, allergies, obesity, and cancer^1,3^. An individual’s gut microbiota composition will vary throughout their lifetime and is dependent upon personal habits such as diet, lifestyle, sterile food consumption, personal hygiene, stress, and antibiotic use^3^.

Probiotics play a crucial role in gut microbiota and there is growing interest among the public and scientific community in using probiotics to potentially regulate the composition of the gut microbiome^1^. Based on the Food and Agriculture Organization of the United Nations (FAO) and the World Health Organization (WHO), probiotics are living microorganisms that provide benefits to the host once they reach sufficient numbers^4^. While there is no uniform recommended therapeutic dosage for probiotic intake, the United States Food and Drug Administration recommends consumption of a minimum concentration of 10^6^ CFU (Colony Forming Units) per mL (or gram) of probiotic viable cells^5^. Currently, probiotics can be found in dairy products such as fermented milk, cheese and yogurt^6^. To expand the probiotic application and to cater to dietary restrictions such as vegan diets and individuals with lactose intolerance, many non-dairy products have been introduced that contain probiotics, for example, juices and cereals^6^. However, research on probiotic encapsulation for use in non-dairy products, in particular raw fruits and vegetables, is limited, in large part, due to the challenges related to the probiotic viability during encapsulation^6,7^. Successful probiotic encapsulation requires consideration of important properties for probiotic viability, including resistance to gastric acidity, storage temperatures, effects of competitive bacteria, and consistency in air and water exposure ^7^.

Another primary motivation is finding more sustainable alternatives for the preservation of fresh produce. Vegetables and fruits are classified as perishable foods: they are sensitive to external conditions and can spoil quickly due to microbial, chemical, or physical actions. Approximately 30% of vegetables and fruits harvested for human consumption are lost due to spoilage, resulting in tremendous waste^1^. The principal objective in food preservation is increasing shelf life while maintaining original color, texture, and freshness under external stresses^8–10^. Traditional food preservation methods including pasteurization, freezing, drying, chilling and chemical preservation may result in nutrient loss and the introduction of undesirable chemicals through enzymatic activity. As a result, there is a need for a natural and green alternatives for fruit and vegetable preservation that both protects and adds nutritional value.

Mucilage, a branch of plant hydrocolloids with a hydrophilic nature, creates a gel-like aqueous solution. Mucilage can be derived from different parts of the plants including seeds, leaves, middle lamella, barks, and root^11^, and are natural blends of several polysaccharide structures with higher swelling ability than the polysaccharides currently used in the pharmaceutical applications^12^. These mucilage based polysaccharide structures provide enhanced barrier properties in environments with low relative humidity, produce slimy masses that take longer to dissolve than currently available natural gums, and can produce durable edible films^13^. Seeds from basil, wild sage, flaxseed, and quince are the most commonly used for mucilage based biodegradable film preparations^11^. Quince seed mucilage is composed of glucuronoxylan which is a natural blend of glucuronic acid and xylose^12^. Flaxseed mucilage is a mixture of arabinoxylan and rhamnogalacturonan I^14^. Basil seed mucilage is a combination of arabinose, xylose, rhamnose, and galacturonic acid^15^. Mucilage-based edible films and coatings are efficient platforms for preserving food quality because of their antimicrobial and antioxidant properties. These cost effective and eco-friendly films and coatings also enhance the appearance of the coated food products^16^. Other advantageous properties of mucilage-based edible thin films include, the formation of inorganic nanoparticles, enhancement of flavors and colors, and the introduction of enzymes, minerals, vitamins, and probiotics to the diet^11,17^. In addition, to their preservation ability these mucilage-based films like other edible films are non-toxic; biocompatible; and can serve as obstacles to the transportation of moisture, oxygen, and aromas. Some of the mucilage extracted from seeds have been used as medical treatments^18,19^. For example, quince seed (*Cydonia oblonga*) is used as a pharyngeal demulcent, in the treatment of stomach ulcers, and for dysentery and diarrhea^11^. Flaxseed, (*Linum usitatissimum),* has been reported to be beneficial for reduction of blood cholesterol and glucose levels^20^. And basil seed (*Ocimum basilicum*) has been traditionally used as a remedy for dyspepsia, ulcers, diarrhea, sore throat, and kidney disease^21^.

The goal of the current study was to develop and characterize green and sustainable probiotic mucilage-based edible films with extra nutritional value which can be used for fruit and vegetable preservation. Several studies have reported the use of probiotics in edible films^22–29^, however, the encapsulation of probiotics into edible mucilage-based films has not been reported to date. These mucilages are natural compatible blends of different polysaccharides with proven medical benefits. We developed eco-friendly and sustainable probiotic-containing natural edible coatings, which reduce fruit waste by keeping them fresh for an extended amount of time. In addition to that, the nature of these coatings suggests extra health benefits provided by the encapsulated probiotics.

## Materials and methods

### Materials

Flax (*L. usitatissimum)* seeds (Arrowhead mills), Basil (*O. basilicum)* seeds (Rani Brand) and the fruits and vegetables (strawberry, banana, cucumber and tomato) used in this study were purchased from a local market in Ithaca, New York. Quince (*C. oblonga)* seeds were purchased from Sadaf Co. (California, USA). The study complies with local and national guidelines. Glycerol (G9012, ≥ 99.5%) and diiodomethane (DIM, ≥ 98%), De Man, Rogosa and Sharpe (MRS) agar and broth, were procured from Sigma-Aldrich (St. Louis, USA). Ethanol (200 proof pure, food-grade) was purchased from Koptec (Pennsylvania, USA). Ultrapure water was used in all steps mentioned and was purified using a Milli-Q (MQ) Integral Water Purification System (Merck Millipore, MA, USA). Phosphate buffer saline (PBS) 100 ml tablets purchased from RPI Research Products International (IL, USA). The bacterial strain *Lactobacillus rhamnosus* GG (LGG) was purchased from Chr Hansen probiotics collection (Denmark). LGG is Gram-positive, rod-shaped, facultative, anaerobic, heterofermentative, lactic acid bacteria, and shows optimal growth at 37 °C.

### Film preparation

#### Flax and quince mucilage preparation

Each seed type was sieved and washed in 100 mL ethanol and stirred for 5 min and decanted to remove surface impurities. Then, any remaining ethanol was allowed to evaporate and the seeds were then dried at 45 °C in an oven overnight. The clean, dry seeds were then presoaked in MQ water for 20 min with a water to seed weight ratio of 25:1^18^. The solution was heated to 80 °C for 20 min during the mucilage extraction to kill potential pathogens^30^. Once the viscosity of the solutions increased, the temperature was lowered to 45 °C and stirring continued for another 5–10 min until a viscous gel-like solution formed. Next, to separate the mucilage from the seeds, the viscous solution was passed through a nylon mesh filter 255 µm (U-CMN-255, Component Supply Co., Tennessee, USA) at a temperature of around 45 °C. Once gels were obtained, under constant stirring (750 rpm), glycerol (5 wt%) was added to the solutions as a plasticizer at 45 °C for 15 min^18,31^. To remove the remaining solid contaminants, the glycerol-gel solutions were poured into Falcone tubes and centrifuged for 15 min at 11,000×g (~ 7000 rpm). Finally, the mucilages were autoclaved at 120 °C for 30 min. The initial weight of seed used for flax and quince mucilage preparation were 18 g and 8 g, respectively.

#### Basil mucilage preparation

10 g of Basil seeds were treated as above until a viscose gel-like solution was formed. Then, the solution was poured the into Falcone tubes, kept at  − 80 °C freezer overnight, and then freeze-dried for 72 h to obtain dried powders^15^. In order to separate the seeds from the mucilage powders, a laboratory sieve where the freeze-dried powders were rubbed against the sieve. To the mucilage powder, MQ water at 80 °C was added with water to seed weight ratio of 25:1 and once the viscosity of the solution increased, heating was stopped and stirring continued for another 5–10 min to form a gel with a high viscosity. Then, similar to previous samples, 5 wt% glycerol was added to the solution constantly stirred at 750 rpm for 15 min at 45 °C. To separate the mucilage from the seeds, the viscous solution was passed through the nylon mesh filter (255 µm) with a temperature of around 45–50 °C and the solution was poured into test-tubes and centrifuged for 15 min at 11,000×g to remove any remaining contaminations^15^. Finally, the mucilage was autoclaved at 120 °C for 30 min.

#### Probiotic mucilage preparation

After preparation of the mucilage samples, the solutions were warmed to 37 °C and 0.1 g of the powdered probiotic LGG added and mixed for 10 min to reach the final concentration of 10^8^–10^9^ CFU/ml.

#### Edible film preparation

To prepare the films, 50 ml of each solution of mucilage, with and without bacteria strain, were poured onto Teflon plates (20 × 10 cm^2^) and dried on leveled trays in a vacuum oven at 37 °C for 48 h. The films were removed and stored for further analysis in resealable plastic bags in the freezer at  − 20 °C to prevent the growth of the bacteria. Before use, the films were kept in the plastic bags until they reached room temperature (25 °C). Non-probiotic films were prepared as a control. The samples without bacteria were named after their seeds (Quince, Flax and Basil) and the samples with bacteria were named with the seed name-B (Quince-B, Flax-B, Basil-B). The digital images of the coated films on the glass slide are shown in Fig. S1.

## Characterization

### FTIR-ATR spectroscopy

The IR spectra of the prepared films obtained using IRAffinity-1S Fourier Transform Infrared Spectroscopy (FTIR) equipped with a Quest® single reflection attenuated total reflectance (ATR) module (Specac, Kent, UK), in the frequency range of 400–4000 cm^−1^ with resolution of 4 cm^−1^ (averaging 128 scans).

### Moisture content, water solubility and activity

The moisture content of the films which is the empty volume in the film's microstructure network filled by water molecules was calculated according to the ASTM D4442 method^32^. Films were dried in an oven at 103 ± 2 °C and their mass change was monitored until a constant weight was obtained.

The water solubility of the films was determined by the ratio of the weighed round-shaped (1 × 1 cm^2^) dry films after immersion in 50 mL of MQ water under constant stirring at 25 °C for 5 h. Then, the films were removed and dried at 100 ± 2 °C until no more change in weight was observed (final dry weight). The solubility percentage (triplicates for each film) was measured using Eq. (1)^17^.

1 $$ \% \;{\text{Solubility}} = \;\;\frac{{\left[ {Initial\; dry\; weight} \right] - \left[ {Final \;dry\; weight} \right] }}{{\left[ {Initial\; dry\; weight} \right] }} \times 100 $$

The water activity of the edible films was measured using AquaLab 4TE water activity meter (METER Group, WA, USA). Each edible film (1.5 × 1.5 cm^2^) was analyzed three times for ~ 5 min with the mean temperature of 25 °C.

A digital micrometer was used to determine the thickness of the films in three different locations and all the measurements were done in triplicate.

### Water vapor permeability

The water vapor permeability (WVP) kinetics of the films was investigated using a modified ASTM E96-95 method^18^. Prepared films were cut and placed in a vial cell with a diameter of 1.5 cm and a depth of 3 cm. To provide a constant relative humidity (RH) of 52% at 25 °C, saturated NaCr_2_O_7_·2H_2_O solution was placed in a desiccator alongside the vial cell^33^. The weight change of the films was examined every 24 h, and the loss in the mass was directly attributed to water evaporation. The results were plotted against time and the slope was normalized to the mass transfer surface area (m^2^) according to (Eq. 2) to calculate WVTR^34^. W is the increase in vial weight during 24 h (t), A is the mass transfer area, X is thickness of the film and ΔP is the saturation vapor pressure of water (Pa) at 25 °C (3.173 kPa). The RH values in desiccator and the vials are R_1_ and R_2_, respectively.

2 $$ {\text{WVP}}\,\, = \;\,\frac{WVTR}{{\Delta P\left( {R_{1} - R_{2} } \right)}} = \frac{{\left( {W \times X} \right)}}{{\left( {A \times t \times \Delta P} \right)}} $$

### Contact angle

To examine the hydrophilicity of the films, contact angle measurement was performed using a Ramé-hart instrument model 190CA (Netcong, NJ) by depositing a small drop (50µL) of MQ water and DIM on films surface. The angle between the film surface was automatically measured via Drop image software V.2.10.02 provided by the manufacturer. The test was done in triplicates and the average was reported.

### UV–Vis spectroscopy

To investigate the behavior of the edible films against visible and UV light, a UV-2600 spectrophotometer (Shimadzu, Japan) equipped with a film holder was used and the samples analyzed in the wavelengths range of 200–800 nm. The transparency of the samples was obtained using Eq. (3)^35^, where the thickness of the films (mm) and transmittance at 600 nm were *x* and *T*_*600*_, respectively. The higher transparency value means the films are opaquer. All tests were done in triplicate.

3 $$ {\text{Transparency}}\;{\text{value}}\;\, = \;\,\frac{{ - log T_{600 } }}{x} $$

### Color characteristics

To measure the color of the films, a chroma meter (Konika Minolta CR-400, Osaka, Japan) and was calibrated by CR-A43 calibration white plate prior to operation^24,30^. Films (~ 1 mm thick) were fixed on glass slides with a white background. The L* (lightness), a* (redness + or greenness-), and b* (yellowness + or blueness-) values of the reflected light from the film surfaces was measured as CIELAB values. The hue angle ($$h$$), chroma ($$C^{*} )$$, and total color difference $$\Delta E$$ were calculated based on Eqs. (4–6)^36^, where Δa*, Δb*, and ΔL* are redness, yellowness intensity are the luminosity difference from the films without bacteria, respectively. At least three measurements were made. The $$\Delta E^{*} < 1$$ means color differences aren’t detectable to the naked eye.$$ 1 < \Delta E^{*} < 3$$ is an indication of minor color differences appreciable by naked eye depending of the hue, and values more than 3 shows the obvious change in human eye^37^.

4 $$ h = \tan^{ - 1} \left( {b^{*} /a^{*} } \right) $$

5 $$ C^{*} = \left( {a^{*2} + b^{*2} } \right)^{1/2} $$

6 $$ \Delta E^{*} = \left( {\Delta L^{{*^{2} }} + \Delta a^{{*^{2} }} + \Delta b^{{*^{2} }} } \right)^{1/2} $$

### Mechanical properties

To investigate mechanical properties of the films, tensile strength and elongation at break were measured according to ASTM D882 using a TA Instruments DMAQ800 at 24 ± 1 °C. Three rectangular films (3 × 1 cm^2^) were mounted between the grips and tested at the crosshead speed of 1 N/s until the samples were ruptured. Tensile Strength (MPa) and Elongation at break are calculated using Eqs. (7) and (8), respectively. In these equations *F*, *w, x*, *L*_*0*_ and *L* were maximum strength of stretching (N), film width and thickness, initial length and lengths at rupture, respectively^17^.

7 $$ {\text{Tensile}}\;{\text{Strength}}\;\left( {{\text{MPa}}} \right) \, = \frac{F}{x \times w} $$

8 $$ {\text{Elongation}}\;{\text{at}}\;{\text{break }}\left( \% \right) \, = \frac{L}{{L_{0} }} \times 100 $$

### Thermal properties

Differential scanning calorimetry (DSC Q2000 TA Instruments, DE, USA) and thermogravimetric analysis (TGA Q500, TA Instruments, DE, USA) were carried out to study the thermal properties and stability of the prepared films. Both DSC and TGA tests were performed at a heating rate of 10 °C/min under N_2_ atmosphere. For the DSC test, in order to erase the thermal history of the films, they were heated from 25 to 150 °C and then cooled to  − 40 °C. At the final heating stage, the samples were heated from  − 40 to 275 °C to investigate their thermal properties. The TGA was conducted in a ramp mode starting from ambient temperature to 500 °C.

### Survival of the probiotics and inactivation rate

The viability of LGG incorporated into the films was done according to colony count technique^30^. In summary, to release the bacteria, 0.1 g of the films containing LGG was gently mixed in 9.9 ml of sterile PBS on a shaker by constant agitation for 1 h. The serial dilutions were cultured on MRS agar and incubated at 37 °C for 48 h. Control samples were also prepared by adding the bacteria to the MQ water. The survivability of the bacteria was assessed by counting the total number of viable bacteria (CFU/g). The logarithmic value of relative viability (log N/N_0_) was used to calculate LGG inactivation kinetics during the storage. As shown in Eq. (9), the viability data were fitted to a first-order reaction kinetics model, in which the number of the viable bacteria at time zero and after a specific time of storage (CFU/g), are N_0_ and N_t,_ respectively. Bacteria storage time in days is represented by, t, and k_T_ is the inactivation rate constant (log CFU/g.day^−1^) at temperature, T^38^.

9 $$ logN_{t} = logN_{0} - k_{T} t $$

### Laser scanning confocal microscopy

Laser scanning confocal microscopy (LSCM) images were taken to visualize the surface roughness and morphology of the prepared films, using a Keyence VK-X260 Profilometer with a 10 × objective in surface profile mode. The images processes using VK-X series multifile analyzer software, where a secant curved surface correction was performed with an auto-adjusted height range.

### Scanning electron microscopy

Scanning electron microscopy (SEM, Zeiss Gemini 500 Field Emission, Germany) images was used to observe the presence and distribution of probiotics on the surface and cross-section of fractured surfaces (using liquid N_2_) of the films at an accelerating voltage of 1.0 kV. Denton Desk V sputter coater (Moorestown, NJ, USA) was utilized to coat the SEM samples with a 15 nm layer of gold–palladium prior the test.

### Statistical analysis

The statistical analysis conducted using Origin software (Version 9, OriginLab, MA, USA) and the results were reported as a mean ± standard deviation (SD). Each dataset was analyzed using one-way ANOVA and *p < 0.05 were deemed to be statistically significant in all of the evaluations.

## Results and discussion

### FTIR-ATR spectroscopy

**Figure 1:**
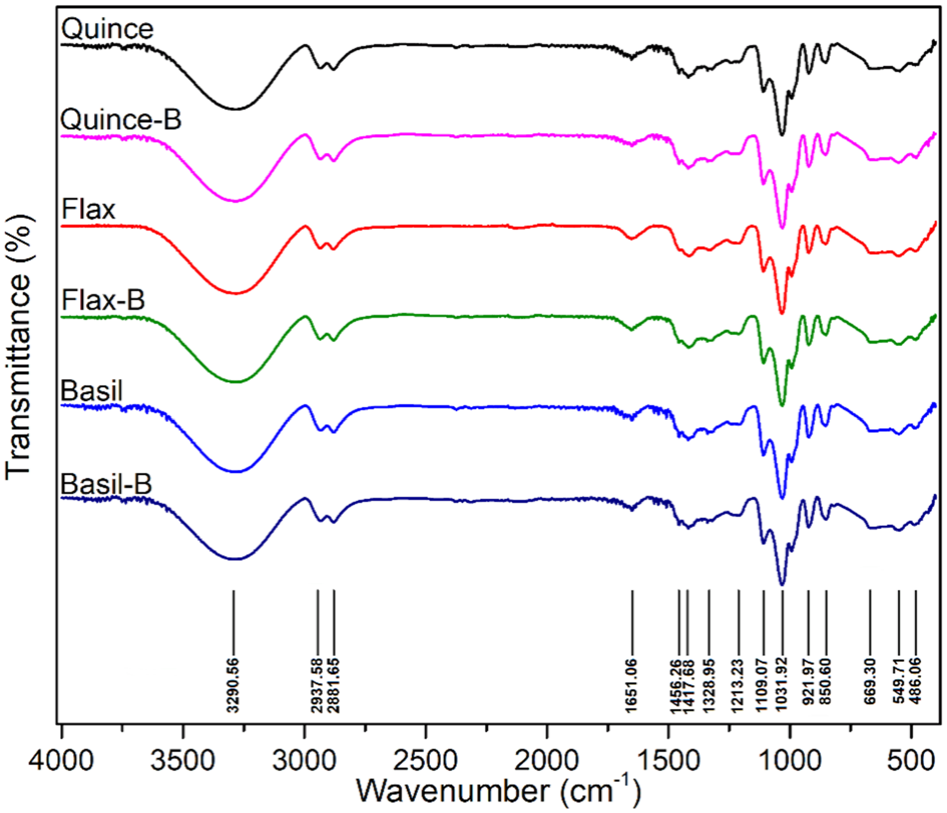
The FTIR spectra of films without LGG and with LGG after 1 month in 4 °C. No noticeable difference was observed between the samples.

The FTIR-ATR spectra of films, with and without probiotic LGG, after 1 month are depicted in Fig. 1.

It can be seen that all the films, obtained from different seed mucilages, show similar polysaccharide structure and the presence of bacteria after one month does not spoil and affect the structure of the samples. Since there was no difference between the FTIR results for the films kept in the fridge and room temperature, we only reported the samples stored in the fridge (4 °C). All samples show characteristic bands between 800–1200 cm^−1^ in the fingerprint region for polysaccharides or 3,6-anhydro-β-galactose^17^. The bands at 1109 and 1031 cm^−1^ belongs to the C–O and –C–O–C glycosidic linkage, respectively. The C–O–O asymmetric and symmetric stretching bands are observed at 1651 and 1417 cm^−1^, respectively^39^. All the samples showed bands at 2937 and 2881 cm^−1^, which are attributed to the methyl symmetric and asymmetric stretching and bending C–H bond vibrations, while the broad band at ~ 3000–3500 cm^−1^ seen in all the samples is indicative of free OH stretching, hydrogen-bonded OH groups and glycerol OH bonds^17,18,40^.

### Thermal properties

To investigate the crystallization behavior and thermal properties of the edible films, a differential scanning calorimetry (DSC) test was performed. It is generally known that the crystallinity can influence the barrier and physical properties of the films including WVP, solubility, and UV transmittance^17,41^. Table 1 summarizes the thermal characteristics of the films.

**Table 1:** Thermal properties of the films with and without probiotic LGG.

Samples	T_g_ (°C)	T_c_ (°C)	WHH (°C)	ΔH_c_ (J/g)	T_i_ (°C)	T_f_ (°C)
Quince	73.82	187.23	35.82	49.60	192.10	366.56
Flax	67.83	208.31	56.71	56.71	211.57	347.01
Basil	71.92	173.70	33.64	47.01	175.55	352.53
Quince-B	74.14	203.69	23.59	37.79	210.46	382.72
Flax-B	68.05	219.67	53.84	44.02	220.49	369.93
Basil-B	72.31	175.62	31.01	22.83	185.11	356.57

The films demonstrated three peaks, typical of DSC plots for polysaccharides. In the first heating, all the samples exhibit an endothermic peak at around 100 °C that can be related to loss of trapped moisture^42,43^ or the heat and water-related phase transitions of the gel structure of the films^44,45^. To confirm that the observed endothermic peak caused by the water vaporization, samples were cooled down to  − 50 °C, and no exothermic peak (cold crystallization peak) of water was observed. The films show glass transition temperature (T_g_) between ~ 67–74 °C depending on the samples as different mucilages have different composition and structures. As shown in Table 1, the presence of bacteria has a slight effect on the T_g_, this confirms that there is no change in the crystalline structure of the mucilage films before the 150 °C upon incorporation of the bacteria.

All the samples demonstrated an endothermic third peak at ~ 173–220 °C that can be attributed to the combustion initiation and sample decomposition (T_c_) at which, glycosidic bonds are cleaved^46^. These films show thermoset-like behavior by having a decomposition peak and leaving char in the DSC pans. It can be hypothesized that heating the bacteria-containing mucilages can kill the bacteria and the available proteins and lipids might be released into the mucilage. According to Knabel, teichoic acids are found in all gram-positive bacteria and are linked to their cell membrane. These acids contain 1,3-poly(glycerol phosphate) or ribitol phosphate and carbohydrates connected via phosphodiester bonds. They also contain glycosyl substituents attached to glycolipids^47^. The presence of these proteins and lipids can act as plasticizers and since we have a high concentration of these bacteria, these plasticizers can affect the crystallinity of the films containing the bacteria. It is generally known that addition of a plasticizer weakens the interaction between molecules and reduces the crystallinity of the resulting polymers^48^*.* As can be seen in Table 1, upon addition of bacteria the enthalpy of crystallization (ΔH_c_) and width of half height crystallization peak (WHH) values decrease in all the mucilage films, which can be due to the release of those glycolipids and a change the microstructure of the crystals. Peak widths that are narrower suggest a slower crystallization rate, however, the increase in the T_c_ of the films after the presence of the bacteria might be due to the fact that these bacteria are negatively charged and can interact with counter ions during the heating process in DSC^47^. It is also reported that teichoic acid can form soluble complexes with polysaccharides in the presence of moderate dielectric constant solvents in the pH range of 4.5–8.2 suggesting that this acid may serve as a complexing agent for hydrophilic molecules^49^.

The thermal stability of edible films was measured by thermogravimetric analysis (TGA) in a nitrogen atmosphere to obtain the degradation starting temperature (T_i_) and final degradation temperature (T_f_) (Fig. S2). All the films show two stage degradation, as the first one belongs to the loss of trapped moisture and the second stage belongs to the decomposition of the glycosidic bonds. It can be seen that upon addition of probiotics both T_i_ and T_f_ have been shifted to the higher temperatures which show that the thermal stability has been increased. Quince based films with and without LGG show higher T_f_ compared to the other two samples which can be due to the occurrence of xylose-uranic acid complexes during the degradation^50,51^. The thermal stability is quite obvious in the first stage as well, since the water loss from the films is slower and the weight loss is decreases upon addition of probiotics especially for the Quince-B sample. The presence of bacteria has decreased the films initial water loss and has increased the films thermal stability. As mentioned earlier this can be related to the bacteria negative charged and their interaction with counter ions during the heating process^47^. Basil and Flax based films show the lowest and highest thermal stability, respectively, although all the films show a similar trend.

### Tensile properties

Tensile strength and elongation at break, are very important mechanical characteristics of edible films and packaging materials. Table 2 shows the tensile properties of the films, in the presence and absence of LGG. Flax films, regardless of the presence of LGG, display increased elasticity with the lowest modulus and highest elongation at break among all the samples. Meanwhile, the basil films show the lowest elongation at break. Interestingly, the quince films show a higher modulus in comparison to flax, however, the tensile strength in flax films is higher. Therefore, out of the three seed mucilage types, quince and flax are expected to be more mechanically robust support materials for the edible film applications.

**Table 2:** Mechanical properties of the films with and without probiotic LGG.

Samples	Tensile strength (MPa)	Elastic modulus (MPa)	Elongation at break (%)
Quince	14.61 ± 0.37^b^	400.63 ± 25.05^a^	45.54 ± 1.36^b^
Flax	16.46 ± 1.08^a^	316.23 ± 18.35^b^	55.68 ± 2.11^a^
Basil	8.53 ± 0.21^c^	294.90 ± 20.25^c^	38.31 ± 0.29^c^
Quince-B	14.13 ± 0.27^b^	422.46 ± 3.81^a^	43.32 ± 0.46^b^
Flax-B	15.87 ± 0.72^a^	346.17 ± 14.11^b^	52.01 ± 1.75^a^
Basil-B	8.04 ± 0.14^d^	301.63 ± 22.30 ^c^	36.11 ± 0.64^d^

^a, b, c, d, e^Different letters in the same column indicate significant differences.

(a > b > c > d; *p* < 0.05). Values were given as mean ± SD.

Upon the addition of LGG to the mucilage films, the tensile strength and elongation at break especially in basil-based films have been decreased since probiotic cells can interrupt the cohesiveness of the polymer chains^30^. The increase in the elastic modulus values for each mucilage after the addition of probiotic LGG is not statistically significant (*p* < 0.05), however, the slight increase might be due to the reduction in molecular mobility and free volume in polymer chains^30,52^. Glycerol was added as a plasticizer to enhance the mechanical properties of the films by decreasing the intermolecular forces between polymer chains and reducing crystallinity^17,30^. Upon the addition of probiotic LGG, the glycerol not only acts as a plasticizer but also provides a better environment for probiotics by reducing the osmotic pressure^53^, which makes the films a suitable platform for preservation of the probiotics. It has been previously reported that addition of lactic acid bacteria in films can improve their mechanical properties especially the elongation at break to a certain extent, as the microbial inclusions might interfere with the plasticizing effect of glycerol^28,54,55^. It is also reported that addition of probiotics in protein-based edible films such as sodium caseinate have changed in the mechanical properties slightly due to the low mass of added probiotics, while cellulose-based edible films are quite sensitive to the addition of probiotics^29,56^. In this study, we observed that upon addition of probiotics the change in mechanical properties is negligible, only basil-based films showed significant changes. The changes in the basil-based films are most likely due to their inhomogeneity or the presence of seed leftovers.

### Water solubility, moisture content, and surface behavior

Thickness of the films is a crucial factor that affects the transparency, water vapor permeability, and mechanical properties of the films. Table 3 summarizes the thickness of the films in the absence and presence of LGG. The results show no difference between the thickness of the samples before and after the addition of LGG, probably because of same amount of solution used for preparation of all the films, as previously reported^57^. In addition, with the same water to seed ratio during the preparation of the films, basil films showed the lowest and flax films showed the highest thickness.

**Table 3:** Thickness, moisture content, water activity, water solubility and contact angle of the films.

Samples	Thickness (µm)	Moisture content (%)	Water activity (-)	Water solubility (%)	θ_m_ in MQW (°)	θ_m_ in DIM (°)	Surface energy (mN/m)
Quince	119 ± 1.0^a^	94.01 ± 1.91^a^	0.431 ± 0.013^a^	52.29 ± 1.04^a^	34.8 ± 5.3^a^	58.2 ± 1.6^c^	62.8 ± 2.6^d^
Flax	125 ± 1.0^b^	94.47 ± 0.04^a^	0.361 ± 0.009^b^	75.01 ± 1.92^c^	26.7 ± 1.6^c^	92.2 ± 4.3^a^	95.3 ± 1.8^a^
Basil	115 ± 1.5^c^	94.08 ± 2.53^a^	0.332 ± 0.008^c^	68.69 ± 2.59^b^	31.1 ± 2.1^c^	77.6 ± 2.9^b^	76.4 ± 2.2^c^
Quince-B	120 ± 0.5^a^	94.55 ± 2.14^a^	0.435 ± 0.011^a^	67.13 ± 2.39^b^	41.6 ± 1.2^a^	77.7 ± 6.8^b^	64.9 ± 1.9^d^
Flax-B	126 ± 0.5^b^	94.09 ± 0.25^a^	0.363 ± 0.010^b^	83.18 ± 2.02^e^	31.7 ± 2.9^c^	94.7 ± 3.6^a^	92.6 ± 0.9^b^
Basil-B	116 ± 0.5^c^	94.64 ± 0.82^a^	0.334 ± 0.011^c^	79.16 ± 1.43^d^	33.9 ± 0.9^b^	81.2 ± 4.4^b^	76.3 ± 2.1^c^

^a, b, c, d, e^Different letters in the same column indicate significant differences.

(a > b > c > d > e; *p* < 0.05). Values were given as mean ± SD.

The moisture content after drying is an important factor in edible films because it can affect the viability kinetics during long periods of storage and facilitate the melting of these edible films inside the oral cavity^58^. The moisture content of the films is reported in Table 3. The films show similar moisture content across all seed types and the addition of probiotics does not have a significant impact on the film’s moisture content (*p* > 0.05). It is noteworthy that in all the samples the presence of glycerol helps maintain the water content and inhibits water evaporation during storage at 4 °C or ambient temperatures, but not at higher temperatures. Moreover, glycerol acts as a humectant providing a suitable environment for the survival of the probiotics^59^.

To address the benefits to food stability, water activity (A_w_) of the films has been measured. A_w_ values close to 1.000 indicate food instability, since the samples can be sensitive to both microbiological (growth of bacteria, yeasts, and mold) and physicochemical changes. However, an A_w_ lower that 0.600 suggests that the films are more stable against microbial growth and will be shelf-stable without any further heat treatment^60^. It can be seen that all the films show low water activity (A_w_ < 0.45) and the quince-based films regardless of the presence of bacteria show the highest water activity. This low water activity inhibits the growth of the bacteria and other microorganisms, while, the addition of glycerol, as mentioned earlier, can protect the water for bacterial survival and prevents the amount of necessary water needed for the survival of the LGG from escaping in the films. The addition of LGG has slightly increased the A_w_ which is related to the pin-holes created on the film`s surface and changes in the surface integrity^30^. Our results are in agreement with previous reports on edible films based on peach^61^ and wine grape pomace^62^ where mucilage-based films were shown to be more stable against microbial growth.

The high moisture content of the films can represent high water solubility of the films. The water solubility of all the films was measured at pH = 7.4, and the results are reported in Table 3. Flax and Quince show the highest and lowest solubility, respectively and upon the addition of LGG the solubility of the films increased. It has been previously reported that the water solubility can be affected by the polarity of the films, water diffusion, ionization of hydroxyl and carboxyl groups, wettability and surface energy, polymer relaxation, and dissociation of hydrogen and ionic bonds^58^ and presence of bacteria might change the wettability, surface energy and water diffusion.

The surface behavior of the films is acquired via contact angle measurement with MQ water and DIM, and the results are summarized in Table 3. To assess film hydrophilic behavior, the contact angle against MQ water is measured. The Quince shows the highest angle, while the Flax shows the lowest values which corroborates the water solubility result which indicated more hydrophilicity in flax-based films. Contact angle changes are function of surface heterogeneity, crystallinity of the polymer, surface energy, and the chemical nature and roughness of the polymer surfaces^63,64^. Upon addition of probiotics, the contact angle values increased which could be due to the increase in roughness of the surface caused by the presence of LGG. It could be expected that the hydrophilicity of the samples increases upon the addition of LGG, however, it seems that an increase in the roughness is more dominant. The DIM results also confirm more hydrophobic behavior for quince-based films compared with the other samples as they show lower values against this organic solvent. Based on contact angles, the surface energy is calculated and interestingly it does not show a similar trend in different samples. The presence of LGG in the films caused a small increase in the surface energy of Quince-B when compared with Quince, which indicates that the presence of bacteria bonds to this film better on the surface. This increase in surface energy can be related to the porous structure caused by the hydrophilic LGG. The surface energy is decreased in Flax-B indicating that LGG weakens the surface bond, although the flax-based films show the highest surface energy regardless of the presence of LGG. Finally, the presence of LGG has not significantly changed the surface energy of basil-based films.

### Water vapor permeability

**Figure 2:**
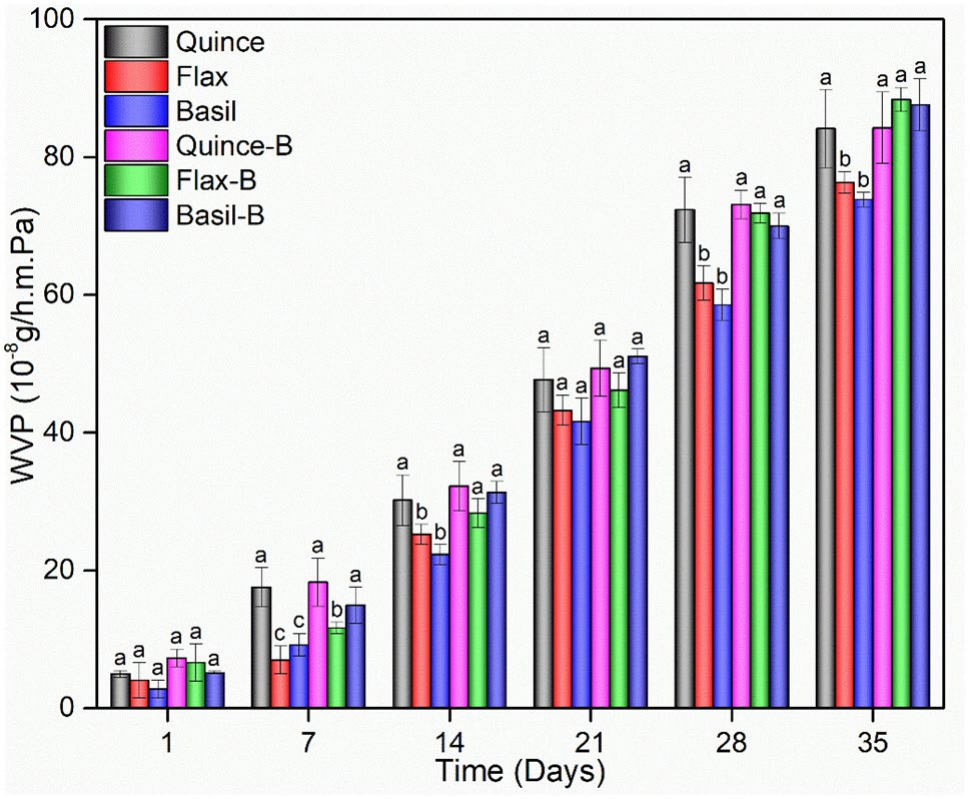
WVP of mucilage films with and without probiotic LGG. Reported values are shown as the mean (n = 3) ± SD. For each set of measurements collected on the same day for each of the films, different letters (a,b,c) within those days indicate a significant difference (a > b > c; *p* < 0.05). From left to right in each group the order is Quince, Flax, Basil, Quince-B, Flax-B and Basil-B.

One of the most crucial properties of edible films is water vapor permeability (WVP), it can be influenced by the integrity and thickness of the films, surface behavior such as hydrophobicity as well as the degree of crystallinity^58^. To maintain the quality of the food, reduction of the moisture transfer between the surrounding environment and the food is essential. Hence, WVP should be kept as low as possible^17^. All the films show an increasing WVP overtime, due to the water saturation of the films and the easier water transfer between the films and environment. however, among the samples without LGG, Basil shows the lowest WVP, while Quince shows the highest value after 5 weeks as shown in Fig. 2.

After the addition of LGG, Initially, films containing LGG show the same trend in which the highest and lowest values belong to Quince-B and Basil-B respectively. Interestingly, initial WVP of the probiotic mucilage films increased by 46, 63, and 85% for Quince-B, Flax-B, and Basil-B samples, respectively compared with the samples without LGG. The increase in WVP is related to the presence of pin-holes created on the surface of the films and changes in the surface integrity due to the incorporation of the probiotics which result in an increase in the moisture absorption^30^. The Flax-B samples show higher values of WVP after 5 weeks, compared to the other samples, which could be due to the thinner films of flax films which produce a porous structure on the surface of films in the presence of LGG. Quince-based films, however, show similar results regardless of LGG presence, which is attributed to the extracted mucilage which is denser and thicker than the others due to the chemical composition and results in more homogenous films. Moreover, according to the observed solubility results, quince-based samples show lower solubility, further evidence that they maintain their integrity and prevent water evaporation.

### Optical properties

Films with reduced UV transmittance can effectively protect food products from unwanted chemical reactions, especially, UV-induced oxidative degradation which results in discoloration, nutrient loss, and off-flavors^17,65^. Therefore, suitable optical properties are one of the most important prerequisites in edible films and food packaging applications, they not only effect consumer preferences but they also maintain the products’ quality^17,57^. There are several important factors that impact the optical properties of the edible films: thickness; crystallinity and mean size of the crystals; structural conformation; compatibility of the film components and plasticizer type and concentration;^57,66^. The UV and visible light transmittance at selected wavelengths (200–800 nm) were measured, and the results summarized in Table 4a and Fig. S3. The addition of LGG caused a significant reduction in transparency (higher opacity) of the films compared with the samples without LGG (*p* < 0.05). It has been previously reported that the presence of probiotic cells in the films can influence light transmission, due to the increase in light scattering^30^.

**Table 4:** Light transmittance, transparency values, and color characteristics.

Films	Wavelength (nm)								Transparency values
	200	280	350	400	500	600	700	800	
**(a) Light transmittance**									
Quince	0.06	0.25	0.13	0.07	0.03	0.02	0.01	0	14.44 ± 0.56^a^
Flax	0.42	1.26	1.36	0.50	0.14	0.06	0.02	0	10.41 ± 0.34^b^
Basil	0.17	0.71	0.48	0.29	0.16	0.09	0.04	0.01	9.11 ± 0.10^c^
Quince-B	0.23	0.88	0.57	0.32	0.15	0.08	0.03	0	9.08 ± 0.27^c^
Flax-B	0.47	1.22	1.12	0.64	0.26	0.13	0.05	0.01	7.01 ± 0.05^d^
Basil-B	0.18	0.69	0.49	0.31	0.16	0.09	0.04	0	9.06 ± 0.06^c^

Films	L*	a*	b*	C*	*H*	ΔE*			
**(b) Color characteristics**									
Quince	83.69 ± 0.02^a^	− 0.69 ± 0.02^d^	13.70 ± 0.04^e^	13.72 ± 0.04^e^	− 1.52 ± 0.01^c^	–			
Flax	84.55 ± 0.01^a^	− 2.24 ± 0.01^f^.	23.48 ± 0.02^c^	23.59 ± 0.02^c^	− 1.48 ± 0.01^c^	–			
Basil	70.39 ± 0.02^b^	2.86 ± 0.02^b^	22.88 ± 0.01^d^	23.06 ± 0.01^c^	1.45 ± 0.01^b^	–			
Quince-B	79.11 ± 0.00^c^	− 0.59 ± 0.01^e^	22.85 ± 0.02^d^	22.86 ± 0.01^d^	− 1.55 ± 0.01^c^	10.23 ± 0.01^b^			
Flax-B	76.13 ± 0.02^d^	− 1.13 ± 0.01^c^	36.65 ± 0.00^a^	36.66 ± 0.01^a^	− 1.54 ± 0.01^a^	15.67 ± 0.02^a^			
Basil-B	64.56 ± 0.04^e^	3.80 ± 0.01^a^	31.27 ± 0.03^b^	31.50 ± 0.02^b^	1.45 ± 0.01^b^	10.25 ± 0.01^b^			

^a, b, c, d, e, f^Different letters in the same column indicate significant differences (a > b > c > d > e > f, *p* < 0.05).

Quince shows the highest transparency values regardless of the presence of the LGG, while Basil shows the lowest transparency. Interestingly the presence of LGG didn`t significantly change the transparency values in Basil films. Moreover, all the samples show low transmission in both UV and visible light regions.

The color characteristics of the edible films are reported in Table 4b. Flax and Quince films show higher luminosity (*L*^∗^), while the Basil has a darker film. It was observed that upon addition of the LGG, the lightness (luminosity) and clearness decreased in all the films. LGG-containing films exhibited higher a* and b* values, which is an indication of more redness and yellowness of the films when compared with the films without LGG. Quince and Flax are greener in color when compared to Basil. The addition of LGG causes the appearance of all the films to become darker with a redder tint which is hard to see by the naked eye. It should be noted that in all the samples, ΔE^*^ values are higher than 3 which is an indication of a visible change, however, the dark shade (chroma) and low hue of the films make the observed changes in color difficult to notice by visual inspection. Overall, throughout the storage period the lightness (*L*^∗^), redness (a^∗^), and yellowness (b^∗^) values of all the samples remained constant (*p* > 0.05, Data not shown).

### Morphology and topography

**Figure 3:**
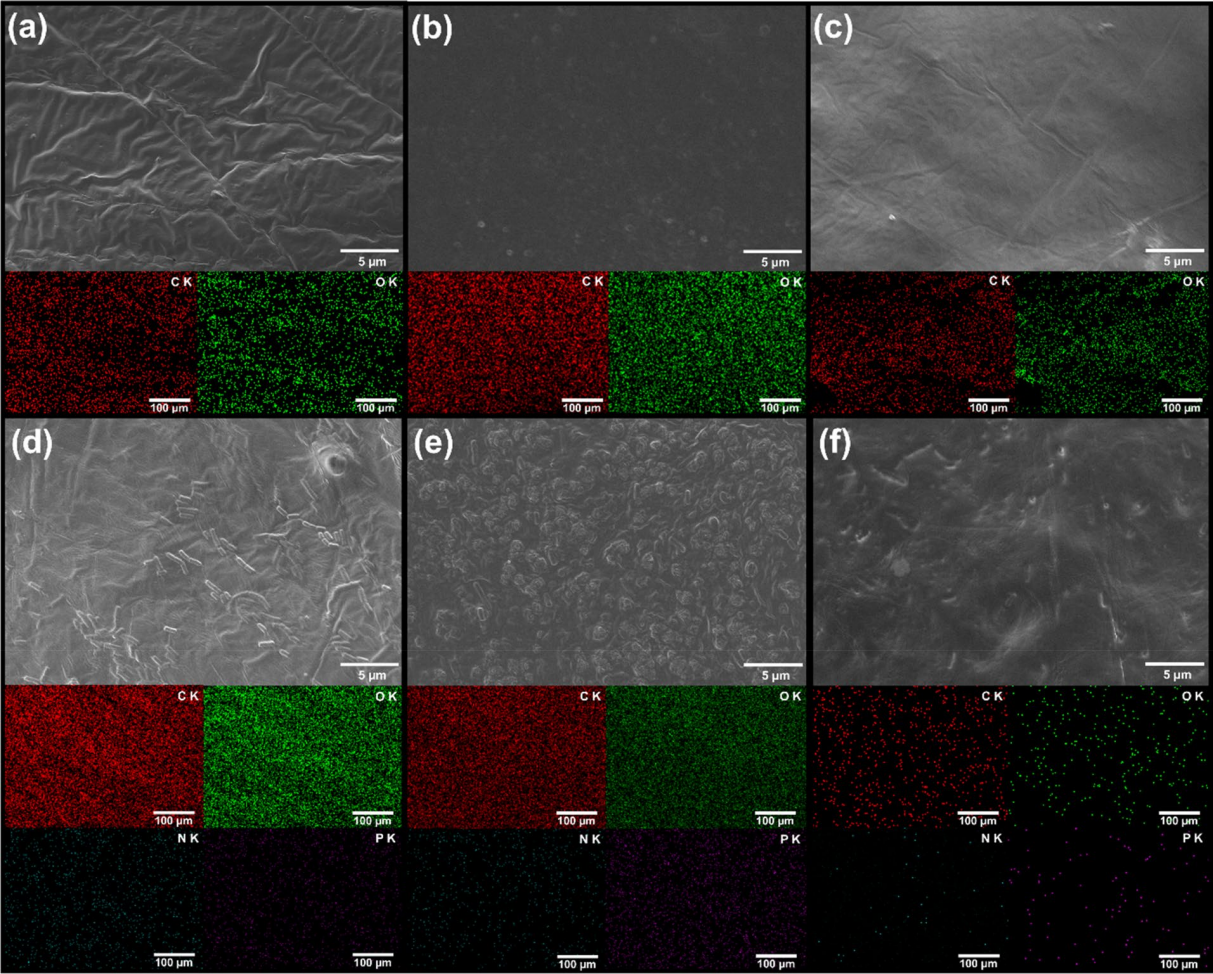
SEM images and elemental mapping from the surface of films (**a**) Quince, (**b**) Flax, (**c**) Basil, (**d**) Quince-B, (**e**) Flax-B, (**f**) Basil-B. The red and green dots represent the presence and dispersion of C and O respectively. In the samples with LGG the in addition to C and O, the blue and purple dots represent the presence of N and P which belongs to amino acids of probiotics.

To observe the morphology of the films and confirm the presence and even dispersion of LGG in the probiotic films, SEM images with EDX (energy dispersive X-ray) were taken of the surface (Fig. 3) and cross-section of the samples (Fig. S4). All the samples show a homogenous surface; however, some samples, especially Quince, show wrinkles on the surface which could be the result of heating the samples (37 °C) to reduce their moisture content during sample preparation for SEM imaging. As mentioned earlier Quince produces a denser and thicker solution when compared with the other two films, which makes the water evaporation in the heating process harder and thus the most noticeable wrinkles when dried. In the films with the probiotic LGG, the rod-like shaped bacteria with a size of ~ 1 μm can be seen. To confirm the homogenous distribution of LGG in films, EDX mapping was used. Every Bacterium strain contains proteins and phospholipids in its structure, therefore, “N” groups (Blue dots) and “P” groups were targeted. The good distribution of N and P groups is a sign of the presence and dispersion of LGG in the films.

**Figure 4:**
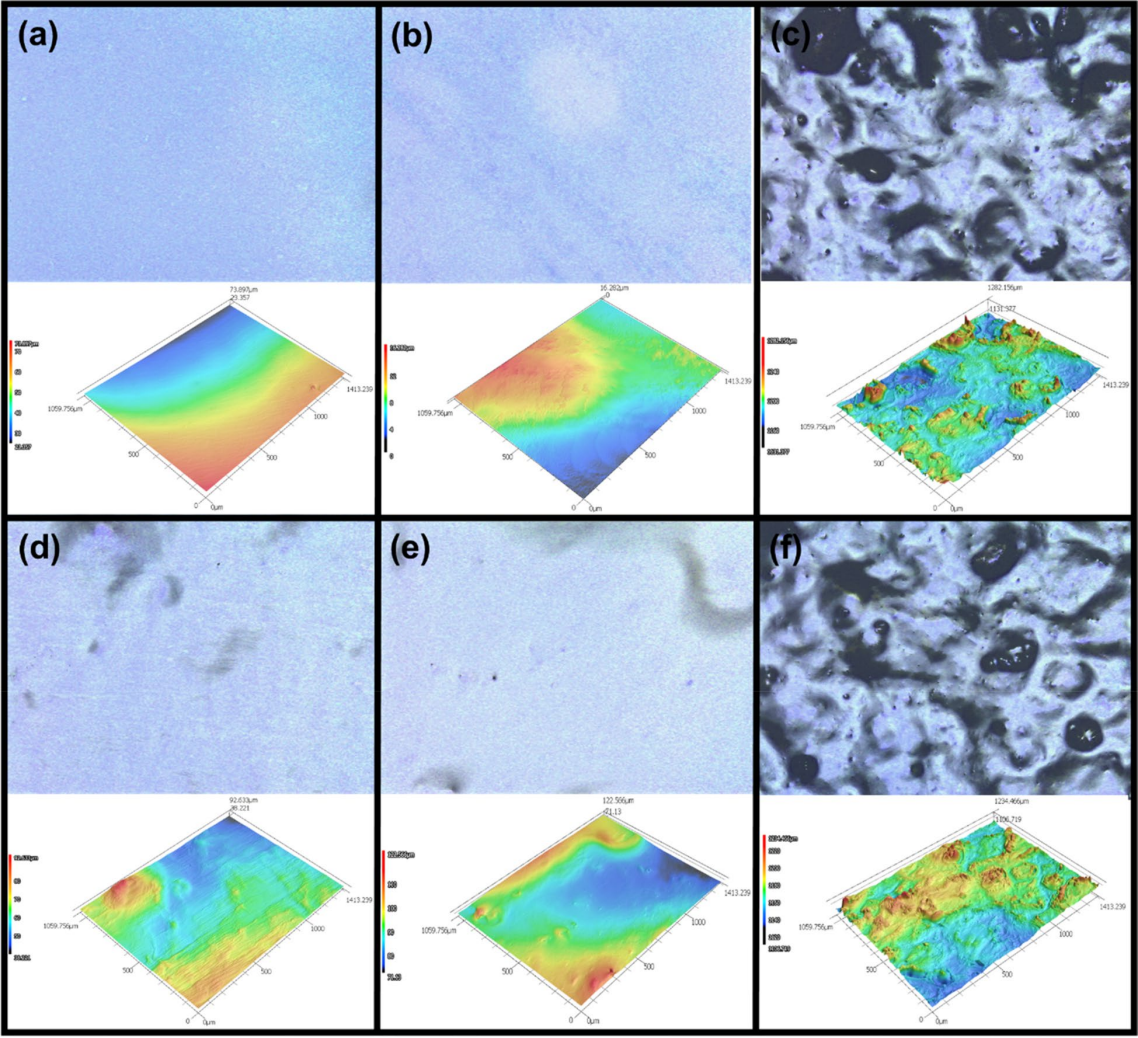
The laser scanning confocal microscopy 2D and 3D images of (**a**) Quince (**b**) Flax and (**c**) Basil, (**d**) Quince-B (**e**) Flax-B and (**f**) Basil-B.

The microstructure and 3D topographical framework of the films were observed using laser scanning confocal microscopy and are demonstrated in Fig. 4. As can be observed in Fig. 4a and b, both Quince and Flax show a smooth surface, while the Basil sample (Fig. 4c) has a rough surface with a porous structure, which could be due to the left-over seed parts that could not be removed from its structure during film preparation. Upon addition of the probiotics, the roughness of the films increased (Fig. 4d, f) compared to the control samples, indicative of the pin-holes caused by the presence of the LGG. The embedment of bacterial cells in the film matrix could result in an increase of the roughness. These results were previously confirmed by SEM and WVP results.

To quantitatively study the surface roughness of the films, average surface roughness (Ra) was measured. Ra values for Quince, Flax, and Basil were found 2.2, 1.6, and 5.5 µm, respectively. Upon the addition of LGG, Ra values for Quince-B, Flax-B, and Basil-B increased to 3.1, 3.9, and 7.6 µm, respectively. This increase is ascribed to the presence of tiny rod-like bacteria embedded in the films. It is important that films do not introduce roughness or color changes to the produce that would discourage consumers. Any increase in the roughness of a film can change the texture and appearance of the coated produce. Quince and flax films are better candidates for edible film applications because they exhibited lower roughness than the Basil films regardless of the presence of LGG.

### Viability of probiotics in films during storage

The viability of the probiotics can be affected by storage temperature, duration, and humidity^67^. Table 5A shows the viability of LGG embedded in films during storage at 4 and 25 °C. All the samples showed no significant decrease in probiotic viability during the drying process (Initial day). As the temperature has a direct effect on the survivability of the bacteria, all the films stored at room temperature showed more reduction in a viable number of probiotics compared with the samples stored at 4 °C (*p* < 0.05). The Quince-B film demonstrated the highest LGG viability among the samples by showing ~ 2 log CFU reduction at the end of storage at 4 °C and ~ 3 log CFU reduction at 25 °C. Based on the viability percentage of Quince-B film, 71 and 61% of the samples survived after 5 weeks of storage at 4 and 25 °C, respectively. Flax-B film shows nearly one more log reduction comparing with the Quince B and the LGG survived in these films after 5 weeks of storage are 61 and 51% at 4 and 25 °C, respectively. Finally, the Basil-B film demonstrates the lowest values of viability by showing nearly 5 (41%) and 7 (19%) log reduction during storage at 4 and 25 °C, respectively. The quince formed a more homogenous film and protected the LGG better comparing with the other two mucilages, while the porous structure of the basil caused lower protection of probiotics.

**Table 5:** Viability of the probiotics during the storage in the films in fridge and room temperature.

Samples	Temperature (^o^C)	Log CFU/g					
		Initial	7 days	14 days	21 days	28 days	35 days
**(A)**							
Control (LGG in MQ)	25	9.06 ± 0.04^a^	8.09 ± 0.05^a^	7.23 ± 0.04^c^	5.30 ± 0.08^d^	3.23 ± 0.45^d^	1.65 ± 0.06^e^
	4	9.06 ± 0.04^a^	8.47 ± 0.04^a^	7.66 ± 0.03^a^	6.06 ± 0.08^c^	5.39 ± 0.12^b^	3.30 ± 0.23^d^
Quince-B	25	8.87 ± 0.09^b^	7.29 ± 0.39^b^	7.42 ± 0.04^b^	7.30 ± 0.24^a^	5.54 ± 0.25^b^	5.47 ± 0.06^b^
	4	8.87 ± 0.09^b^	8.57 ± 0.09^a^	7.67 ± 0.07^a^	7.52 ± 0.03^a^	6.41 ± 0.27^a^	6.36 ± 0.16^a^
Flax-B	25	8.89 ± 0.01^b^	7.54 ± 0.10^b^	7.56 ± 0.25^b^	7.13 ± 0.03^b^	5.81 ± 0.07^b^	4.56 ± 0.05^c^
	4	8.89 ± 0.01^b^	8.54 ± 0.41^a^	8.21 ± 0.40^a^	7.65 ± 0.18^a^	5.73 ± 0.29^b^	5.39 ± 0.23^b^
Basil-B	25	8.58 ± 0.10^c^	6.30 ± 0.59^b^	5.75 ± 0.01^e^	4.90 ± 0.07^e^	2.79 ± 0.24^e^	1.68 ± 0.09^e^
	4	8.58 ± 0.10^c^	7.17 ± 0.48^b^	6.80 ± 0.16^d^	5.15 ± 0.04^d^	4.32 ± 0.27^c^	3.60 ± 0.13^d^

Samples	Inactivation rate at 4 °C k_T4_ (log CFU/g day^−1^)	R^2^	Inactivation rate at 25 °C k_T25_ (log CFU/g day^−1^)	R^2^			
**(B)**							
Quince-B	0.078^c^	0.941	0.081^c^	0.893			
Flax-B	0.108^b^	0.913	0.111^b^	0.902			
Basil-B	0.143^a^	0.976	0.187^a^	0.963			

^a, b, c, d, e^Different letters in the same column indicate significant differences.

(a > b > c > d > e; *p* < 0.05). Values were given as mean ± SD.

The inactivation curves of LGG incorporated into the mucilage based edible films are obtained by plotting the Log(N/N_0_) vs time. The inactivation rate, as well as the R^2^ coefficient, are shown in Table 5B. In all of the samples, regardless of the storage temperature, inactivation of LGG followed first-order kinetics**.** It is also observed that the samples show a slightly higher inactivation rate upon storage at 4 °C. Interestingly no significant differences in the stability of LGG in the Quince-B and Flax-B at different temperatures were observed, and Quince-B showed the lowest inactivation rate compared to the other samples because the water activity of this sample is slightly higher than other films. Basil-B, as expected, based on the viability tests, shows the highest inactivation of probiotics at different temperature (*p* < 0.05).

### Coating applications

Edible films were applied to the surface of samples via a common dip-coating method, to study the effect of films on the shelf life of vegetables and fruits. Among the three seed mucilage types tested, flax and quince were observed to be the more optimal materials due to having better physical and mechanical properties, therefore for a qualitative coating application, we coated produce with Quince, Flax, Quince-B and Flax-B. Prior to the coating process, fresh strawberries, bananas, cucumbers, and cherry tomatoes were plunged in sodium hypochlorite (1%) for 15 min, washed with MQ water and left in the room temperature to dry for 2 h. Then the vegetables and fruits dipped into the as-prepared mucilage aqueous solution for 2–3 min and then air dried^17,68^.

**Figure 5:**
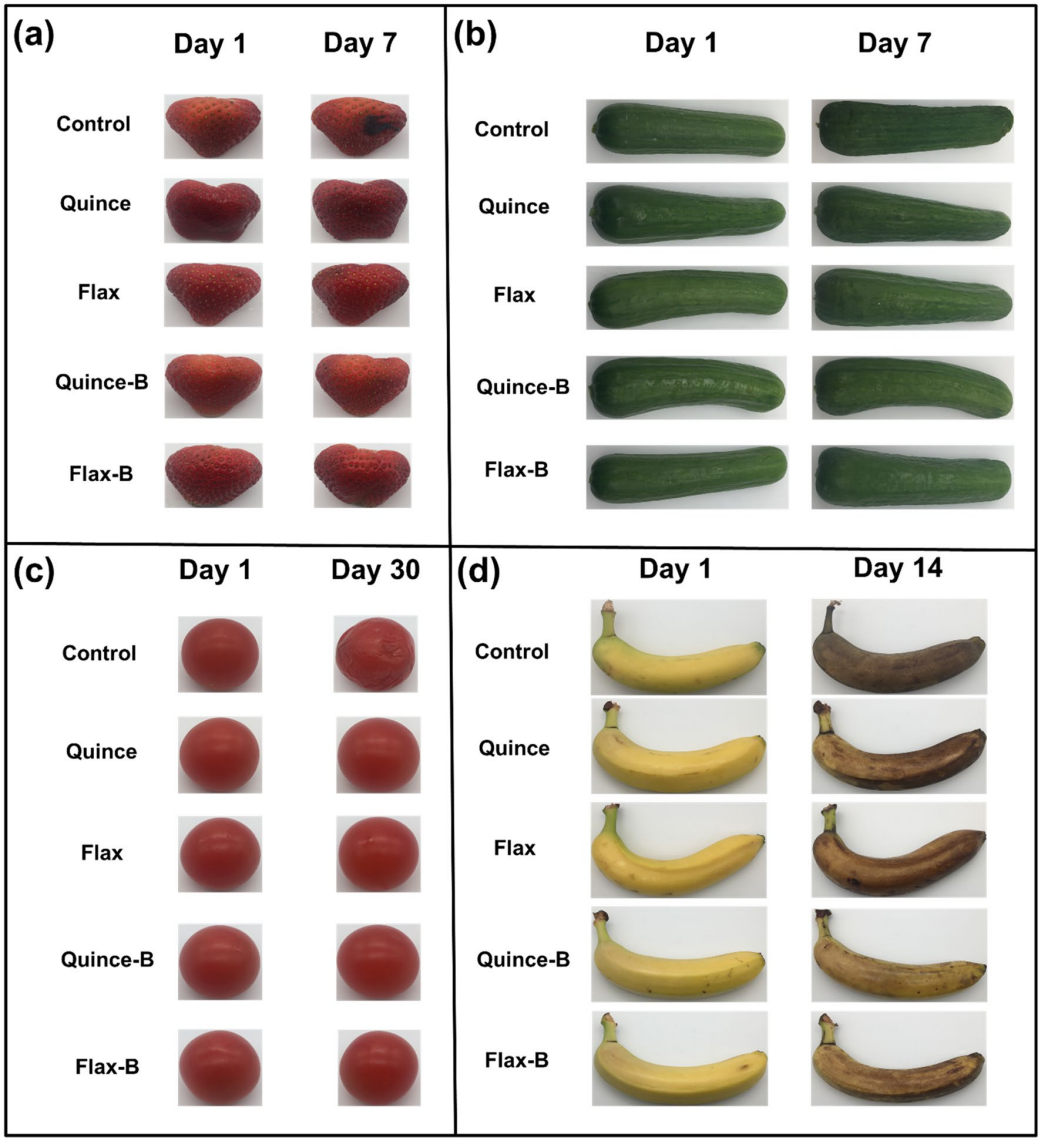
(**a**) Strawberries, (**b**) cucumbers, (**c**) cherry tomatoes, and (**d**) banana coated with quince and flax with and without probiotic LGG as compared with the uncoated samples.

As shown in Fig. 5a, mold formed on the surface of the uncoated strawberries, which were stored in the fridge (~ 4 °C), within a week. The mucilage coated strawberries, however, with all of the films, were brighter and showed lower water loss compared with the control. The presence of LGG preserved the fruit’s original appearance after one week.

In the case of cucumbers and cherry tomatoes (Fig. 5b,c), water loss was the major phenomenon in the studied period (7 days for cucumber and 30 days for cherry tomatoes). The tomato and cucumber appearances were slightly shiny in mucilage coated samples as compared with the control. After 1 month of storage, the tomato`s water loss is readily observable in the control sample, while the appearance of the mucilage-coated samples remained unchanged. For cucumbers, after 7 days, the control sample showed evidence of dehydration (wrinkled surface), while the Quince and Flax coated samples show only a slight water loss. The cucumbers coated with the bacteria-containing mucilage showed almost no change after 7 days.

The banana samples (Fig. 5d) however, showed a visible difference between all the samples. The control sample was completely brown after two weeks, while the banana coated with the films were noticeably less brown. Further the Quince film coated banana was less brown when compared with the banana coated with the Flax film. This could be due to the fact that the films can protect the surface from oxidation which result in discoloration^17,65^. This is more interesting when it is observed that the samples coated with the Quince-B and Flax-B show even less brownish coloration (oxidation), which could be attributed to the activity of the bacteria as it inhibits metabolite formation.

Overall, there was no major difference in the results of the most of the products coated with different mucilage films, except for the banana samples. The mucilage-coated samples retained a better appearance, indicating that the prepared films have a protective effect. Furthermore, it has been reported that the presence of lactic acid bacteria in films not only boosts health when they are consumed but also they can improve protective abilities by competing with other bacteria and pathogens for nutrients alongside producing organic acids and bacteriocins as metabolites during storage^69^. Therefore, products coated by mucilage containing LGG films remained fresher for a longer time.

## Conclusion

In the current study, edible films and coatings based on natural mucilage with lactic acid probiotic strain LGG were fabricated and fully characterized. The addition of LGG to the films had a minor impact on their physiochemical properties. All the films show hydrophilic properties and upon addition of LGG, hydrophilicity decreased. The films showed similar moisture content and water activity regardless of the seed type and presence of probiotics, while the addition of LGG increased the water-solubility of the films and surface roughness. According to the observed solubility results, quince-based films show lower solubility and less change in WVP as they could maintain their structure and prevent water evaporation. Quince-based films show the highest transparency values and surface energy regardless of the presence of the LGG and addition of LGG decreased transparency. The Quince-B film showed the highest LGG viability among the samples by showing ~ 71% and 61% viability of the LGG after 5 weeks of storage at 4 °C and 25 °C, respectively. Finally, the potential application of the prepared films was investigated in a coating experiment on fruits and vegetables. Quince-B showed the best results in all the coatings compared with the other samples. The results of this study indicate that seed mucilage, especially quince, is a viable support material to encapsulate probiotics and preserve food quality. Therefore, mucilage of various parts of the plants such as leaves, middle lamella, barks, and root should also be investigated as source materials for edible thin films capable of encapsulating probiotics. Being able to use them for this application helps divert waste materials and yield value-added products. The probiotic containing edible-thin films reported here are 100% natural and are superior candidates for sustainable food coatings applications.

## Supplementary Information

Supplementary Information.
