# Supplementary material for: Development and characterization of probiotic mucilage based edible films for the preservation of fruits and vegetables
Source: Sci Rep. 2021 Aug 16;11:16608. doi: 10.1038/s41598-021-95994-5 (PMC8368057; doi:10.1038/s41598-021-95994-5)
Supplement: Supplementary file 1 — Supplementary Information. [file 41598_2021_95994_MOESM1_ESM.docx]

**Supporting Information**

Development and Characterization of Probiotic Mucilage Based Edible Films for the Preservation of Fruits and Vegetables

Seyed Mohammad Davachi^1^, Neethu Pottackal^2^, Hooman Torabi^1^, Alireza Abbaspourrad^1*^

**^1^** Department of Food Science, College of Agriculture & Life Sciences, Cornell University, Stocking Hall, Ithaca, New York, 14853, United States

**^2^** Department of Materials Science and Engineering, College of Engineering, Cornell University, Bard Hall, New York, 14853, United States

^*^Corresponding Author. Email address: [Alireza@cornell.edu](mailto:Alireza@cornell.edu) (A. Abbaspourrad)


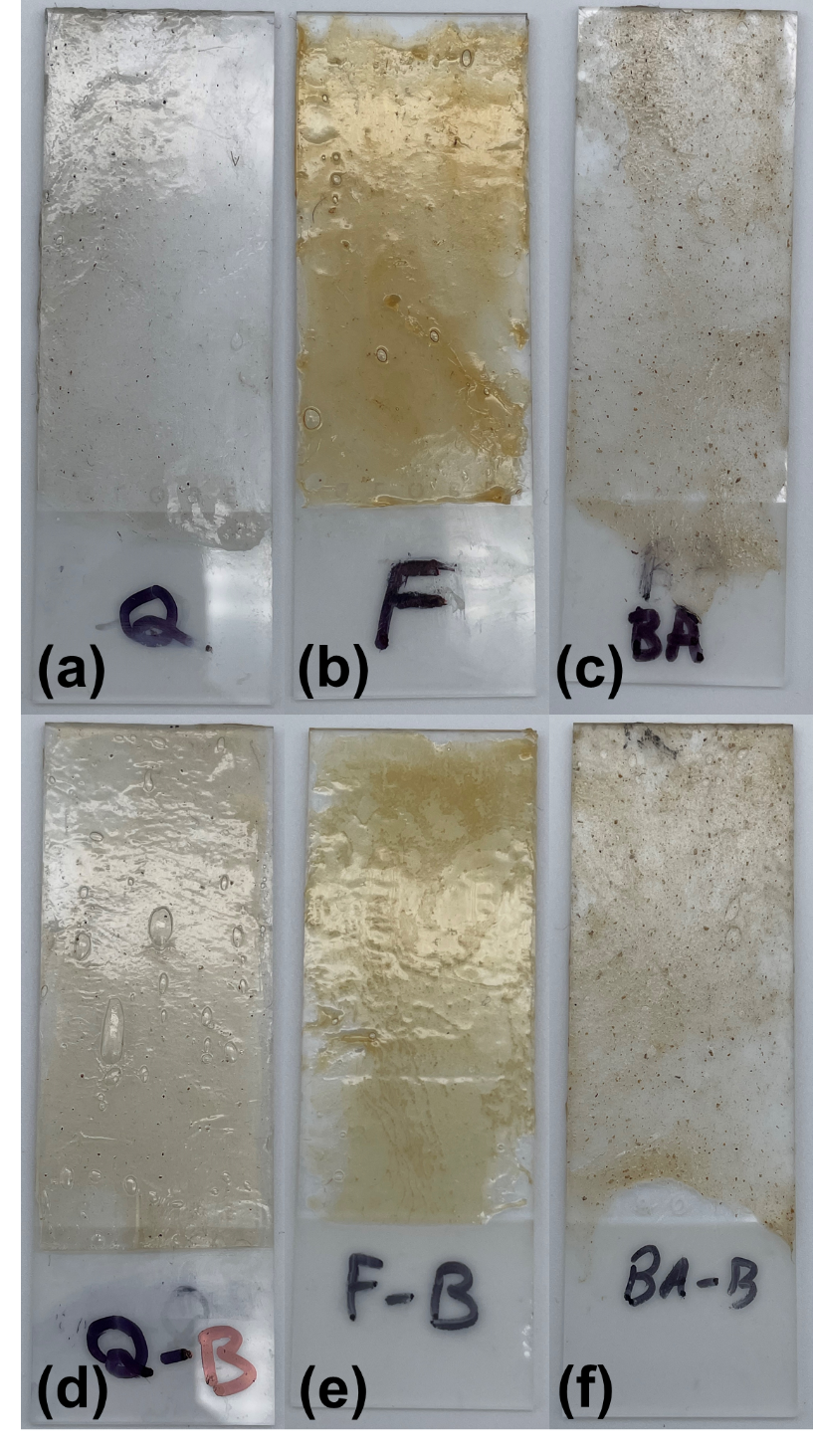


**Figure S1.** Digital images of the films coated on the glass slide (slightly thick for better observation)





**Figure S2.** TGA thermograms of edible films with and without probiotics at rate of 10^o^C/min





**Figure S3.** The UV-vis spectra for all the samples within the wavelength range of 200-800 nm.


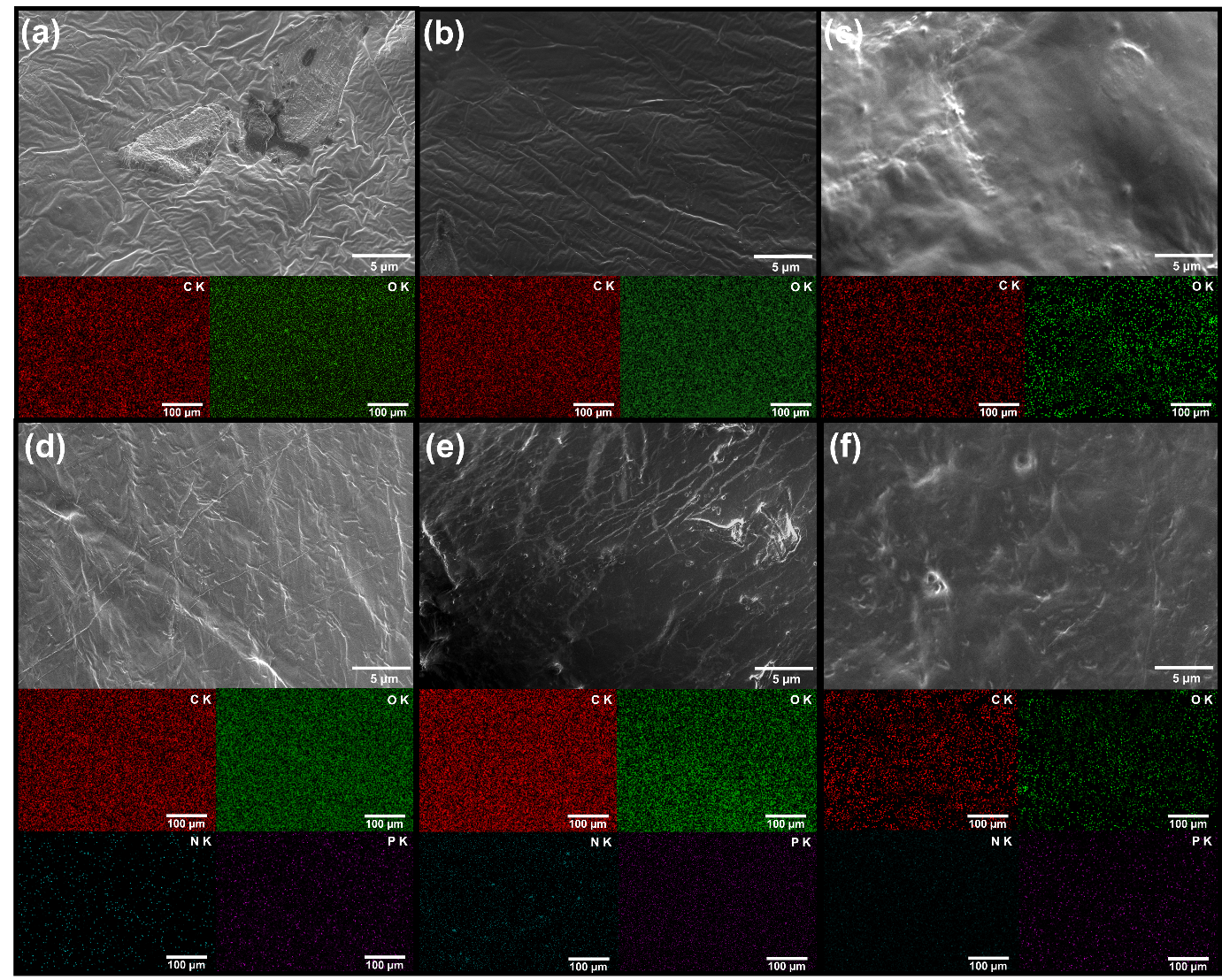


**Figure S4.** SEM images and elemental mapping from the cross-section of the films (a) Quince, (b) Flax, (c) Basil, (d) Quince-B, (e) Flax-B, (f) Basil-B. The red and green dots represent the presence and dispersion of C and O respectively. In the samples with LGG the in addition to C and O, the blue and purple dots represent the presence of N and P which belongs to amino acids of probiotics.
